# Association Between Preoperative Negative Emotional States and Eye Movement During Photorefractive Keratectomy

**DOI:** 10.18502/jovr.v20.17300

**Published:** 2025-12-08

**Authors:** Hesam Hashemian, Hooman Ahmadzadeh, Alireza Razavi, Hassan Asadignadomani, Zahra Montazeriani, Sogol Olamazadeh, Mehdi Khodaparast

**Affiliations:** ^1^Farabi Eye Hospital, Tehran University of Medical Sciences, Tehran, Iran; ^2^Research Center for Clinical Virology, Tehran University of Medical Sciences, Tehran, Iran; ^3^Translational Ophthalmology Research Center, Farabi Eye Hospital, Tehran University of Medical Science, Tehran, Iran; ^4^Department of Medical Physics and Biomedical Engineering, Tehran University of Medical Sciences and Advanced Medical Technologies and Equipment Institute (AMTEI), Tehran, Iran

**Keywords:** Anxiety, DASS-21, Depression, Photorefractive Keratectomy, Stress

## Abstract

**Purpose:**

To describe the link between negative emotions—depression, anxiety, and stress—and eye movement during photorefractive keratectomy (PRK).

**Methods:**

This comparative case series was conducted on 53 PRK candidates and involved completing the Depression, Anxiety, and Stress Scale (DASS-21) before surgery. Eye movement, measured as the radial distance between the pupil and laser center during each shot, was analyzed. Average distance indicated centralization accuracy, while standard deviation (SD) indicated precision. Stress, depression, anxiety, and their relationship with eye movement during PRK were studied.

**Results:**

The mean and SD of eye movements were not significantly correlated with depression, anxiety, stress, or the total DASS-21 score. A weak negative correlation was observed between the preoperative spherical equivalent (SE) and stress (*r* = –0.305, *P* = 0.004), anxiety (*r* = –0.401, *P* < 0.001), and total DASS-21 scores (*r* = –0.324, *P* = 0.002). Lastly, a weak positive correlation was found between ablation time and the SD of eye movement (*r* = 0.473, *P* < 0.001).

**Conclusion:**

The DASS-21 questionnaire showed no link between negative emotions and eye movement. Additionally, longer ablation times correlated with greater SD of eye movement.

##  INTRODUCTION 

Uncorrected refractive errors are the most important cause of visual impairment worldwide.^[1]^ As an alternative to glasses or contact lenses for optical correction of refractive errors, laser corneal refractive surgery has grown in popularity.^[2]^ Despite years of continuous advancements in refractive surgery techniques, photorefractive keratectomy (PRK) remains a highly common refractive procedure worldwide.^[3,4]^ The disadvantages of PRK consist of a slower healing rate, risk of haziness, and longer healing process. At the same time, its advantages include a more residual stromal bed and a reduced risk of ectasia.^[3]^

Before surgery, most patients experience some degree of anxiety and stress, which is a natural reaction to unpredictable and risky conditions. Nevertheless, excessive stress and anxiety levels can potentially have a negative impact on treatment outcomes.^[5]^ Since surgery is a complex procedure, we are unable to review the moderators of intraoperative stress and how they affect performance and outcomes.^[6]^ Accordingly, some studies investigated the patient's cooperation during the PRK surgery as a crucial factor for its success.^[7]^ This is because stress and anxiety can affect the patient's cooperation during surgery. It is possible to experience eye and head movements during the procedure as a result of stress, which can manifest differently in each eye.^[7]^ In this context, we decided to investigate the effect of depression, anxiety, and stress on the amount of eye movement during PRK using the Depression, Anxiety, and Stress Scale (DASS-21).

##  METHODS

### Study Design and Patients

This comparative case series enrolled patients from the Cornea Clinic at Farabi Eye Hospital. Participants signed informed consent and completed eye examinations from January to March 2023. The examinations included uncorrected visual acuity (UCVA) and best-corrected visual acuity (BCVA) using E-chart (converted to LogMAR), manifest and cycloplegic refraction, intraocular pressure examination, slit lamp evaluation, complete posterior segment assessment, and Pentacam tomography (Oculus Optikgeräte GmbH, Wetzlar, Germany). Patients filled out the DASS-21 questionnaire to assess depression, anxiety, and stress 15-30 minutes before PRK. A single skilled surgeon performed all procedures, including PRK, in which the epithelium was removed with an alcohol application for 20 seconds. Eye tracking was centered on the entrance pupil for laser ablation using Amaris 1050RS (SCHWIND eye-tech-solutions, Kleinostheim, Germany). The Schwind excimer laser uses real-time eye-tracking technology to guarantee accurate ablation.^[8,9,10]^ Before the commencement of the ablation procedure, the patient is instructed to focus on a designated target. At this point, the Schwind eye tracker captures the positions of critical ocular landmarks, such as the center of the pupil and Purkinje reflections. Throughout the ablation process, a high-speed camera, along with an analysis processor, continuously monitors the eye, identifying any movements or deviations from the initial calibration position. Surgery data were extracted directly from the device upon completion. The study aimed to investigate the correlation between preoperative emotional states (as measured by the DASS-21) and eye movements. The sample size was calculated to be at least 39 patients, considering a 15% effect size, 95% confidence level, and 80% power using Cohen's formula. This study received approval from the Ethics Committee (ID: IR.TUMS.FARABIH.REC.1401.047) and the Institutional Review Board of Tehran University of Medical Sciences. The study was conducted in accordance with the principles outlined in the Declaration of Helsinki. All patients provided signed informed consent.

### Eligibility Criteria 

The study included patients over 18 years of age who had a maximum keratometry of < 47 diopters (ranging from 37.00 to 47.00 diopters based on the study by Alsheri et al^[11]^) and a regular Pentacam reading. Individuals with any type of eye disease, including keratoconus, corneal scar, history of hydrops; those with a previous eye surgery, including corneal cross-linking, corneal inlay, and keratorefractive surgery; those not completing the follow-up; and those with a corneal thickness < 470 micrometers (to minimize the possibility of developing post-refractive surgery ectasia) were excluded from the study. We also excluded patients with poor cooperation or recognized difficulties before surgery, such as narrow palpebral fissures or suspicious topography.

### Data Collection Instruments

#### Depression, Anxiety, and Stress Scale (DASS-21)

DASS-21 is a self-assessment tool with three subscales to measure negative emotional states: depression, stress, and anxiety. Each subscale has seven items. The depression subscale assesses dysphoria, hopelessness, and lack of interest. The stress subscale evaluates autonomic arousal and situational anxiety. The anxiety subscale measures difficulty relaxing and nervous arousal. Examples include: *"*I felt like I had nothing to look forward to*"* (Depression), *"*I found it difficult to wind down*"* (Stress), and *"*I experienced trembling in the hands*"* (Anxiety).^[12]^

Sahebi et al translated this questionnaire into Persian, and its reliability and validity were confirmed.^[13]^ The Persian version of the DASS-21 has demonstrated excellent psychometric properties, making it an effective and reliable assessment tool.^[16]^ This makes it an invaluable resource for accurately evaluating depression, anxiety, and stress levels among the Iranian population.^[14]^

In our study, participants rated their experience of each condition over the past week on a 4-point scale from 0 (not at all) to 3 (very much). The scores for depression, stress, and anxiety were obtained by summing the respective scores.^[15,16]^ The responses to the component items were added up to determine the scores for each subscale. Participants were given a clear explanation of the study's purpose and instructions at the beginning. Only those who confirmed their willingness to participate were given access to the questionnaire. Responses were structured with fixed choices, requiring participants to complete all questions to avoid missing data.

#### Mean and standard deviation (SD) of eye movement 

In our work, we used the data provided by the laser eye tracker to measure eye movement, defined as the radial distance between the pupil and laser centers, based on the location of the pupil center in each laser shot during the operation. The mean of these values can be considered an indicator of accuracy in centralization. In other words, a lower mean value represents less eye movement during ablation. Moreover, the SD of pupil center locations data, which indicates how spread-out the data is compared to the average value, was determined by taking the square root of the sum of eigenvalues as a measure of centering accuracy. It means the lower the SD value, the more precise the centralization is.

**Table 1:** Basic characteristics of study patients undergoing PRK

**Variables**	**Mean ± SD**	**Min**	**Max**
Age	31.55 ± 7.89	19	55
Mean of eye movement ( μ m)	127.53 ± 74.73	9	463
SD of eye movement ( μ m)	200.05 ± 68.91	68	401
UCVA (post-op) (log MAR)	0.25 ± 0.31	0.1	1.0
BCVA (pre-op) (log MAR)	0.02 ± 0.04	0.0	0.2
Ablation time (s)	14.85 ± 6.67	2.00	32
Spherical equivalent (D)	-2.71 ± 1.81	-8	4
Depression score	2.75 ± 2.79	0	9
Anxiety score	2.75 ± 2.42	0	10
Stress score	5.28 ± 3.13	0	12
DASS total score	10.79 ± 7.21	0	28
DASS, Depression Anxiety Stress Scales; SD, standard deviation.			

**Table 2:** Correlation of variables of depression, anxiety, stress, and DASS 21 total score with mean and standard deviation (SD) of eye movement, spherical equivalent, sex, and age

**Variable**	**Correlation**	**Depression score**	**Anxiety score**	**Stress score**	**Total score**
Mean of eye movement	*r*	0.013	0.116	0.104	0.089
	*P*-value	0.897	0.245	0.298	0.373
SD of eye movement	*r*	–0.033	0.159	0.074	0.072
	*P*-value	0.741	0.108	0.461	0.467
Spherical equivalent	*r*	–0.148	–0.401	–0.305	–0.324
	*P*-value	0.171	< 0.001	0.004	0.002
Sphere	*r*	–0.088	–0.411	–0.271	–0.290
	*P*-value	0.417	< 0.001	0.011	0.006
Cylinder	*r*	–0.190	–0.020	–0.119	–0.132
	*P*-value	0.078	0.855	0.272	0.224
Sex	*r*	–0.200	–0.066	–0.111	–0.148
	*P*-value	0.040	0.499	0.256	0.130
Age	*r*	–0.125	–0.060	–0.194	–0.153
	*P*-value	0.202	0.541	0.046	0.118
SD, standard deviation.					

**Table 3:** Correlation of variables of spherical equivalent, ablation time, sex, and age with the mean of eye movement and the SD of eye movement

**Variable**	**Correlation**	**Mean of eye movement**	**SD of eye movement**
Spherical equivalent	*r*	–0.116	–0.109
	*P*-value	0.283	0.314
Sphere	*r*	–0.150	–0.086
	*P*-value	0.164	0.427
Cylinder	*r*	0.096	–0.083
	*P*-value	0.375	0.447
Ablation time	*r*	–0.113	0.473
	*P*-value	0.302	< 0.001
Sex	*r*	–0.015	0.023
	*P*-value	0.878	0.815
Age	*r*	–0.051	–0.211
	*P*-value	0.611	0.032
SD, standard deviation.			

### Statistical Analysis 

Continuous variables were assessed for normality by the Kolmogorov-Smirnov test and reported as mean ± SD when normally distributed, or as median ± interquartile range otherwise. Qualitative values were reported as frequency (percentage). Pearson's correlation coefficient (*r*) was used to measure the statistical association between variables if normality could be assumed, and Spearman's rank correlation was applied otherwise. Paired *t*-test was used to compare two related samples to identify significant differences in case of normal distribution, whereas a Wilcoxon signed-rank test was used if the data lacked normal distribution. For data analysis, SPSS version 22.0 (IBM Inc., Chicago, IL, USA) was used at a significance level of 0.05.

##  RESULTS 

This study analyzed data from 103 eyes of 53 patients (34 females and 19 males) with a mean age of 31.55 ± 7.89 years (range, 19-55 years). The mean ablation time was 6.67 ± 14.85 seconds (range, 2-32 seconds). The preoperative UCVA averaged 0.25 ± 0.31 (range, 0.1-1.0 LogMAR [logarithm of the Minimum Angle of Resolution]), while the postoperative BCVA improved to 0.02 ± 0.04 (range, 0.0-0.2 LogMAR). The mean preoperative spherical equivalent (SE) was –2.71 ± 1.81 diopters (range, –8.0 to 4.0 D). Preoperative scores for depression (D), anxiety (A), stress (S), and the total DASS-21 score were 2.75 ± 2.79, 2.75 ± 2.42, 5.28 ± 3.13, and 10.79 ± 7.21, respectively. The mean eye movement was 127.53 ± 74.73 (range, 9-463), and the SD of eye movement was 200.05 ± 68.91 (range, 68-401) [Table 1].

Postoperative visual acuity (VA) showed significant improvement (*P* < 0.001). Eye movement metrics were not correlated with depression, anxiety, stress, or total DASS-21 scores (*P* > 0.1 for all). Subgroup analyses based on DASS-21 severity levels (normal to extremely severe) confirmed no significant correlations between the mean or SD of eye movement and psychological scores. Similarly, no correlations were observed between SD of eye movement and depression, anxiety, or stress scores (*P* > 0.1 for all). A weak negative correlation was observed between anxiety/stress scores and sphere [Table 2].

Ablation time showed a positive correlation with the SD of eye movement (*r* = 0.473, *P* < 0.001) [Table 3]. UCVA showed no significant correlation with either the mean or SD of eye movement (*P* = 0.388 and *P* = 0.848, respectively). Gender also did not correlate with these eye movement measures (*P* = 0.878 and *P* = 0.815, respectively). Age exhibited a weak negative correlation with the SD of eye movement (*r* = –0.211, *P* = 0.032), but it was not associated with the mean eye movement (*P* = 0.611). A weak negative correlation was noted between anxiety/stress scores and SE [Table 2]. The correlations between sphere and cylinder variables and both the mean and SD of eye movement were weak and not statistically significant [Table 3].

##  DISCUSSION

This study was designed and implemented to investigate the association between the level of negative emotional states recorded in the DASS-21 questionnaire, as well as its subscales, with eye movement in patients who were candidates for PRK. The results of this study indicate that patients with lower SE exhibit higher anxiety scores, and a weak negative association exists between these two variables.

As a result of variance in pupil center position, the mean of eye movement may not entirely represent the actual amount of eye movement during PRK. As part of our investigation, we calculated the SD of the distance between the pupil center location and the mean pupil movement at each laser pulse for each patient during ablation. In this study, a moderately positive correlation was found between ablation time and the SD of eye movement. Our findings suggest that the increase in the ablation time may lead to a significant increase in the SD of eye movement. Indeed, a large SD of eye movement indicates that patients experience greater and more dispersed eye movements during ablation than their mean pupil movement. A previous study has shown that patients with lower VA generally indicate greater decentration during surgery. It was also postulated that these patients cannot fixate on the distant blinking light.^[8]^ In our sample, SE and UCVA were not significantly correlated with eye movement measures. However, higher eye movement was observed in patients with higher ablation time. Longer ablation times with more laser shots provide more opportunities for patients' eye movement, resulting in higher decentration.^[17]^

Research has demonstrated that elevated anxiety levels in patients can adversely affect the outcomes of refractive surgeries such as PRK.^[18,19]^ Anxiety often leads to increased involuntary eye movements during the procedure, which can prolong the ablation process and diminish the precision of the laser treatment.^[18,19]^ To address these challenges, techniques such as offering a fixation target, utilizing a soft bandage contact lens, and administering mild sedatives can be effective in minimizing eye movement and enhancing surgical outcomes.^[19]^

Our results demonstrated a notable improvement in VA following the procedure. Research indicates that patients generally experience enhanced VA after undergoing PRK, with many achieving 20/20 vision or better.^[20]^ Another study highlighted that PRK delivered excellent refractive outcomes and long-term stability, with the majority of patients achieving 20/20 vision or better 12 months after surgery.^[20]^

Some reports have shown that body position can affect eye movements during PRK. A study with customized ablation showed that most rotational movements (in 46 eyes) were caused by positional changes, and these movements were more significant between upright and supine positions.^[21]^ The supine position may show higher cyclotorsions due to vestibular input variations.^[9]^ As a result of these cyclotorsions, laser ablation can be misplaced, resulting in irregular astigmatism and poor VA correction without recovery.^[22]^ In the present study, we investigated the effect of emotional states on eye movements during the PRK procedure. We did not find a significant association between DASS-21 emotional states and eye movement measures. However, it is worth noting that most of our patients had low preoperative scores, which may have obscured any existing effects. Eye movement during PRK can negatively affect the aberration and contrast sensitivity outcomes and result in decreased VA. Therefore, it is essential to identify patient groups with a high propensity for eye movement. Studying specific populations with high levels of anxiety and stress would be beneficial in this respect.

There are key differences between conventional PRK and transepithelial PRK (trans-PRK). Trans-PRK typically results in faster visual recovery, with patients achieving better VA sooner and experiencing less post-surgical pain.^[19,23]^ The epithelial layer heals more quickly in trans-PRK.^[19,23]^ Both procedures effectively correct low to moderate myopia and astigmatism and offer comparable refractive outcomes.^[23]^ Conventional PRK involves manually removing the epithelial layer before reshaping the cornea, while trans-PRK combines both steps using a laser, which may prolong the procedure.^[24]^ This extended treatment time can lead to potential complications, such as loss of centration, although modern techniques have reduced these risks.^[24]^

Emotional factors play a crucial role in the outcomes of refractive procedures such as SMILE (small incision lenticule extraction) and femto-LASIK (femtosecond laser-assisted in situ keratomileusis).^[25]^ Research has shown that patients' psychological well-being can significantly influence their perception of surgical outcomes and overall satisfaction.^[25,26,27]^ For instance, preoperative concerns such as anxiety, depression, and unrealistic expectations can affect how individuals view their outcomes and their quality of life following surgery.^[26,28]^ Conversely, patients who maintain a positive emotional state and possess realistic expectations tend to report higher levels of satisfaction and better results.^[29]^ Additionally, emotional support and a strong doctor–patient relationship can greatly enhance both the surgical experience and recovery process.^[30]^ By addressing emotional concerns and providing comprehensive counseling before and after the procedure, we can significantly improve patient outcomes and overall satisfaction.

Uncooperative patients undergoing SMILE or femto-LASIK procedures can experience intraoperative complications. A major concern is suction loss, which occurs when a patient moves or fails to adhere to instructions.^[31]^ This issue is particularly pronounced in SMILE due to longer laser cutting time and lower suction pressure, sometimes necessitating the abandonment of the procedure. Complications may include incomplete lenticule dissection, decentration, or interface abnormalities, all of which can compromise visual outcomes. Excessive eye movement may also lead to difficulties in laser centration, increasing the risk of postoperative aberrations and suboptimal refractive results.^[32,33]^

With faster ablation speeds and advanced tracking systems, modern excimer laser platforms have significantly improved refractive surgery, particularly for patients with less compliant eyes. These developments reduce the time patients must stay motionless during treatments, which is particularly important for individuals who may have fixation issues.^[34]^ Reduced anxiety and greater patient satisfaction have been linked to shorter treatment durations. Furthermore, real-time eye-tracking systems enable constant laser monitoring and modification, reducing the impact of eye movements and guaranteeing more precise corneal reshaping.^[34]^ Older excimer lasers, on the other hand, had less sophisticated tracking and slower ablation speeds, which resulted in longer surgery times and a higher chance of misalignment.^[35,36]^ This could have a detrimental effect on safety and visual results in less cooperative patients.^[36]^

This study had some limitations. First, some data might be missing because this is a retrospective study. The small sample size limited the analysis. Additionally, the study did not include an evaluation of the accuracy of the laser center and pupil center alignment by the surgeon prior to commencing the surgical operation. It is imperative to take into account the correlation between the data collected from both eyes while merging them. Disregarding this correlation can lead to flawed interpretations that can have serious consequences. High skewness in ablation time warrants caution in interpreting results. Acknowledging skewness as a limitation enhances the rigor of research. It is crucial to note that this study did not evaluate the quality of vision, specifically factors such as contrast sensitivity and higher-order aberrations. This oversight may significantly impact the overall assessment. To better understand the progression of VA and refractive stability, we must extend the follow-up period for a more comprehensive evaluation. In this regard, it is essential to explore the distinct patterns of eye movements observed between the first and second eye. Analyzing these differences can provide valuable insights into visual processing and coordination. It would be intriguing to investigate the potential correlation between patients' motivation and their SE. It is rewarding to conduct larger, well-designed studies with longer follow-up periods to evaluate the effect of negative emotional states on eye movement before PRK.

In summary, no association was found between eye movements and negative emotional states, as measured by the DASS-21 questionnaire. Lower SE in PRK candidates slightly increased anxiety in these patients. An increase in ablation time was associated with an increase in the SD of eye movement. Future studies on patients with specifically high DASS-21 scores (with high stress, anxiety, and depression) would help identify patient groups with an increased risk of eye movement before PRK.

##  Financial Support and Sponsorship

None.

##  Conflicts of Interest

None.
